# Novel Developments in the Treatment of Multiple Myeloma-Associated Bone Disease

**DOI:** 10.3390/cancers15235585

**Published:** 2023-11-25

**Authors:** Martin Johansen, Mette Bøegh Levring, Kasper Stokbro, Marta Diaz-delCastillo, Abdul Ahad Khan, Line Adsbøll Wickstroem, Michael Tveden Gundesen, Ida Bruun Kristensen, Charlotte Guldborg Nyvold, Mikkel Østerheden Andersen, Thomas Levin Andersen, Niels Abildgaard, Thomas Lund

**Affiliations:** 1 Department of Hematology, Odense University Hospital, 5000 Odense, Denmark; martin.johansen3@rsyd.dk (M.J.); ida.bruun.kristensen@rsyd.dk (I.B.K.);; 2 Department of Clinical Research, University of Southern Denmark, 5000 Odense, Denmarkkasper.stokbro@rsyd.dk (K.S.);; 3 Department of Oral and Maxillofacial Surgery, Odense University Hospital, 5000 Odense, Denmark; abdul.ahad.khan@rsyd.dk; 4 Department of Forensic Medicine, Aarhus University, 8200 Aarhus, Denmark; 5 Center for Spine Surgery and Research, Lillebaelt Hospital, 5500 Middelfart, Denmark; 6 Research Unit for Hematology & Pathology, Hematology-Pathology Research Laboratory, University of Southern Denmark & Odense University Hospital, 5000 Odense, Denmark; 7 Department of Pathology, Odense University Hospital, 5000 Odense, Denmark

**Keywords:** multiple myeloma, kyphoplasty, vertebroplasty, osteonecrosis of the jaw, antiresorptive agents, bone marrow microenvironment

## Abstract

**Simple Summary:**

Multiple myeloma is the second most common hematological malignancy, and the majority of patients have osteolytic lesions by the time of diagnosis. Bone destruction increases the risk of fractures and spinal cord compression, reduces quality of life, and is associated with increased mortality. This paper focuses on current and novel medical and surgical treatment modalities and improvements in prevention and the treatment of therapy-related complications, in particular, medication-related osteonecrosis of the jaw. A special focus reviews new promising targets in the bone marrow microenvironment.

**Abstract:**

Osteolytic bone disease is present in about 80% of patients with multiple myeloma at the time of diagnosis. Managing bone disease in patients with multiple myeloma is a challenge and requires a multi-faceted treatment approach with medication, surgery, and radiation. The established treatments with intravenous or subcutaneous antiresorptives can cause debilitating adverse events for patients, mainly osteonecrosis of the jaw, which, traditionally, has been difficult to manage. Now, oral surgery is recommended and proven successful in 60–85% of patients. Patients with spinal involvement may benefit from surgery in the form of vertebroplasty and kyphoplasty for pain relief, improved mobility, and reestablished sagittal balance, as well as the restoration of vertebral height. These procedures are considered safe, but the full therapeutic impact needs to be investigated further. Ixazomib, the first oral proteasome inhibitor, increases osteoblast differentiation, and recently published preliminary results in patients treated with Ixazomib maintenance have promisingly shown increased trabecular volume caused by prolonged bone formation activity. Other novel potential treatment strategies are discussed as well.

## 1. Introduction

Multiple myeloma (MM), a plasma cell cancer, is the second most common hematological malignancy [1,2], characterized by the proliferation and expansion of monoclonal plasma cells in the bone marrow. Osteolytic lesions are a hallmark finding in patients with MM, and as many as 80% of patients have osteolytic bone disease at the time of diagnosis [3]. The increased degradation of bone causes a high risk of skeletal-related events (SRE], such as pathological fractures and spinal cord compression, and greatly contributes to morbidity [4]. The burden of symptoms in patients with MM has a high impact on health-related quality of life (HRQoL), mainly due to reduced physical function and bone pain. Compared to other hematological malignancies, the disease is associated with a higher risk for patients to end up receiving disability pensions [5,6,7]. Moreover, SRE in patients with MM is associated with inferior overall survival [8]. Thus, managing bone disease in patients with MM is important. In addition to antineoplastic treatment, the treatment of myeloma bone disease may involve radiotherapy, antiresorptives, and percutaneous orthopedic interventions such as vertebroplasty and kyphoplasty, as well as major surgery. The aim of this manuscript is to review the literature and practices of antiresorptive treatments, their adverse effects, and the management of these, along with current standards and new developments in vertebroplasty and kyphoplasty, including examples of bone reconstructive surgery. Antineoplastic treatment and radiotherapy are not within the scope of this review; instead, we focus on potential novel targets in the bone marrow microenvironment.

## 2. Antiresorptive Treatments

In a healthy individual, bone homeostasis is maintained through the continuous resorption of old bone, which is followed by the coupled refilling (formation) of new bone within the bone resorption cavity. Myeloma bone disease arises in patients with MM due to uncoupled bone remodeling with upregulated osteoclast and downregulated osteoblast activity [9,10,11]. The receptor activator of nuclear factor-κB (RANK), a transmembrane receptor expressed in several bone cells as well as in hematopoietic osteoclast precursor cells, activates upon binding with RANK-Ligand (RANKL) and facilitates pre-osteoclast recruitment and osteoclast activation and survival [12]. Myeloma plasma cells increase RANKL expression and decrease osteoprotegerin (OPG) expression. OPG is a decoy receptor that inhibits bone resorption by binding to RANKL and prevents it from binding to its receptor, RANK [13]. The increased RANKL to OPG ratio favors the activation of osteoclasts [14]. The cornerstone in the treatment of MBD is antiresorptive (AR) medication, which inhibits osteoclast activity. Amino-bisphosphonates, zoledronic acid (ZA), and pamidronate (PA), specifically, have for several years been the standard in the treatment of MBD [15]. ZA has the highest relative potency of all bisphosphonates, which is a hundred times higher than that of PA [16]. Either one is recommended as a first choice for all patients with active MM, with or without radiological findings of MBD [17], because micro-architectural changes can be present at the earliest stages of the disease [18]. ZA is preferred, partly because of its more convenient administration time, as well as its superiority over PA regarding mortality rate reduction [19,20]. ZA has shown a progression-free survival (PFS) benefit [21], is not inferior to PA in reducing SREs or bone pain [22], and is superior in treating malignancy-related hypercalcemia [23]. 

After the discovery of the RANKL pathway, denosumab (Dmab), a human immunoglobulin G2 anti-RANKL antibody, was developed. It inhibits RANKL and is the preferred choice when bisphosphonates are contraindicated (e.g., renal impairment). It is speculated that Dmab could be a viable replacement for bisphosphonates in the treatment of MBD. A large double-blind, double-dummy, randomized trial comparing Dmab to ZA that included 1718 patients, however, found no difference in the median time to the first on-study skeletal-related event and showed no significant difference in incidence for serious adverse events, such as medication-related osteonecrosis of the jaw (MRONJ) [24]. An exploratory end-point was later published from the same study. It found that treatment with Dmab resulted in an increased median PFS by 10.7 months, but only in patients intent on undergoing autologous stem cell transplantation and patients with CrCl > 60 mL/min [25]. Future prospective studies are needed to validate this finding. As of 2021, The International Myeloma Working Group recommends ZA over Dmab until more data are available [17]. So far, Dmab has been shown to be as effective as ZA in preventing MBD, but it comes at a higher cost. Furthermore, the later discontinuation of Dmab may be more troublesome than the discontinuation of ZA as bone resorption may rebound. This phenomenon will be discussed in detail in a later section. A study is currently investigating if Dmab can effectively delay the time for high-risk smoldering myeloma to transform into MM requiring active treatment (clinicaltrials.gov: NCT03792763, accessed on 1 November 2023), and data are awaited. The recent approval of romosozumab (anti-sclerostin antibody) for the treatment of osteoporotic patients is a promising development that may translate into treatment for patients with MM. It is supported by pre-clinical observations that sclerostin inhibition prevents fractures and pathological bone loss in patients with MM [26,27,28].

## 3. Medication-Related Osteonecrosis of the Jaw and Multiple Myeloma

The jaws are rarely symptomatically affected directly by the cancer as much as the spine and hip bones. However, the high-dose AR treatment involves a risk of developing MRONJ. One nationwide population-based cohort study in Denmark found the incidence of MRONJ to be around 2% in patients treated with high-dose AR [29]. Other studies have found ZA MRONJ incidence to be from 2.6% to 4% [20,30]. Importantly, however, is that the incidence of MRONJ rises with higher cumulative doses of, or treatment duration with, AR therapy [31]. One systematic review and meta-analysis including a total of 42.003 patients with different malignancies showed that with 4 mg of ZA every 3 or 4 weeks, MRONJ incidence was 2.0% versus 1% when ZA was given every third month. MRONJ incidence with 120 mg Dmab given every month was 2.09%, whereas it was zero when 60 mg was given every 6 months [32]. However, in the currently recommended treatment regimes in MM, 120 mg Dmab or 4 mg ZA both given every four weeks, a large randomized phase 3 study including only MM patients found the risk of developing MRONJ to be similar between the two treatments [24].

The precise mechanism behind MRONJ development is not fully understood, but the development is closely associated with dental extraction or pressure damage from oral prostheses [33]. Theories involve micro-trauma or infection in the bone, triggering an impaired bone healing process, which leads to inflammation with compromised blood supply and subsequent bone necrosis. Recent studies indicate that infection probably is the major determinant of MRONJ [34,35,36,37] and, thus, eliminating local infection may prevent the development of MRONJ. Dental extraction and minor oral surgeries can safely be performed with minimal risk of MRONJ when performed in combination with prophylactic antibiotic therapy [38,39,40]. Additional risk factors include smoking and diabetes as these also compromise healing and increase susceptibility to oral infections after treatment. Prevention and risk reduction of developing MRONJ include appropriate dental treatment before initiating antiresorptive therapy, close monitoring during treatment, and regular dental evaluations to prevent developing dental infections following AR initiation [41].

Temporary discontinuation of AR treatment before upcoming oral surgery, known as a drug holiday, has been considered as a strategy to minimize the risk of MRONJ. However, a large meta-analysis including a total of 6808 patients found no significant difference in the development of MRONJ between the drug holiday group (*n* = 4847) and the control group (*n* = 1961) [42]. One RCT included 23 patients undergoing surgical tooth extraction and randomly allocated patients to a drug holiday from 1 month prior to 3 months post-surgery, with the majority of patients receiving Dmab. It also found no evidence of drug holidays preventing MRONJ. They did, however, report a decline in the patient-reported health state in those on a drug holiday compared to those in the drug-continuation arm [43]. 

Diagnosing MRONJ is based on radiological and clinical findings, including exposed necrotic bone and fistulas to underlying necrotic bone, with symptoms such as jaw pain, non-healing ulcers, and soft tissue swelling. Radiological imaging can help distinguish MRONJ from other oral pathologies and help stage the amount of necrosis for subsequent treatment planning. Conservative treatment was previously recommended for patients with MM who developed MRONJ [44] as this was believed to slow or stop the progression of osteonecrosis. This may include antibiotic therapy, local oral hygiene maintenance with chlorhexidine, the removal of sequestered bone, or minor debridement of the site [44]. However, a recent study shows that despite conservative treatment, MRONJ still progresses in 80% of patients with stage I MRONJ [45]. Thus, the European task force on MRONJ now recommends surgical treatment, when possible, but conservative treatment may still be indicated in frail, elderly patients or in a palliative setting [33]. 

**Figure 1:**
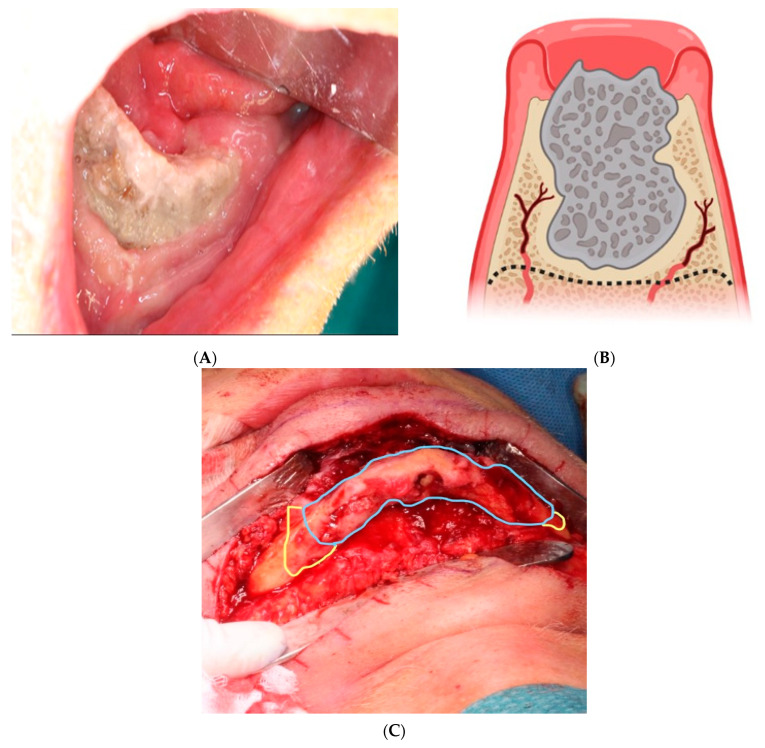
(**A**) Clinical presentation of medication-related osteonecrosis of the jaw (MRONJ). The stage 3 osteonecrosis in the anterior part of the mandible is clinically visible as grey bone protruding through the inflamed gingiva. (**B**) MRONJ. The figure shows the area of necrosis and the suggested resection area for resection. The edges are slightly rounded to avoid sharp edges that may perforate the gingiva during healing. (**C**) Surgical treatment with submandibular incision to access the necrotic bone. A wide incision is necessary to ensure that all necrotic bone is removed, and the mandible can subsequently be restored by a reconstruction plate. Blue line marks the extention of visible osteonecrosis. Yellow line marks the intermediate, sclerotic zone.

Recommended surgical treatment (Figure 1) consists of resection of the osteonecrotic tissue and primary closure of the gingiva. The procedure is planned from a 3D cone beam or multislice CT scan. Surgical treatment is performed in combination with antibiotic therapy (amoxicillin with clavulanic acid, 3 g/day, a minimum of one day before and 6–9 days after surgery) [46]. An incision is made that extends from the perforation of the osteonecrosis in both mesial and distal directions on the alveolar ridge. The gingiva is carefully elevated to access the osteonecrotic tissue and the bone is resected with a drill to the planned extension and depth. The remaining bone should display vital bleeding points. Biopsies can be taken from the vital bone and from the resected necrotic bone to confirm the diagnosis of MRONJ, eliminate an additional cancer diagnosis, and confirm vital bone in the margins of the resection. Finally, the gingiva is mobilized and sutured to a tension-free primary closure. In case the gingiva cannot close the defect, a cutaneous flap must be raised to close it. Surgical treatment has been shown to successfully remove necrosis and infection in more than 60 to 85% of treatments [47]. To increase the chance of successful surgical treatment, adjuncts to surgery, such as teriparatide [48,49] platelet-rich fibrin/plasma [50], and growth factors [51], have shown promising results, but require further investigation. Additionally, fluorescence-guided surgery also shows an increase in success rates by ensuring that all the infected and necrotic bone is removed [52,53]. Overall, the level of evidence for optimal treatment is low for all treatment protocols, building on few randomized controlled trials [37]. The preferable and most effective measure seems to be the prevention of MRONJ by preventing infection and inflammation in the bone by dental examination at 3-month intervals [39].

## 4. Antiresorptive Agents and Duration of Treatment

Before the emergence of MRONJ, AR treatment usually continued indefinitely. However, since incidences of both MRONJ and atypical femur fractures [54,55] increase with the dose and duration of AR treatment, guidelines have been updated to reflect this and reduce its usage. Since the original studies with ZA and PA had a follow-up of approximately 2 years, this is what most guidelines recommend [19,56]. The British Myeloma IX study, however, found that the increased protective effect of ZA compared to the inferior clodronate remained significant beyond 2 years of treatment. Likewise, a Mexican study found a 20% reduction in SRE in patients with MM receiving ZA for 4 years compared to only 2 years [57]. A recent presentation at IMW 2023 presented data from the Magnolia trial, a randomized study comparing 2 vs. 4 years of treatment with ZA in patients with MM, found that the risk of progressive bone disease (PBD) was significantly lower in the 4-year ZOL arm, with a hazard ratio of 0.38, without an increased significant risk of MRONJ [58]. To mitigate the risk of side effects with prolonged AR treatment, some guidelines suggest that the treating physician can consider decreasing the dosing frequency after 12 months of treatment to every 3 months for patients obtaining VGPR or better [15]. This is mainly based on the Myloma IX study, which found that the positive effect of ZA over clodronate on PBD disappeared in patients that had obtained CR + 100 days after autologous stem cell transplantation [59]. However, clinical studies have also demonstrated that patients obtaining a deep response post-transplant have a very low risk of future PBD [60]. Two studies including mainly patients with other malignant diseases than MM found treatment with ZA every 12 weeks versus every 4 weeks to be non-inferior; in these studies, the 12-week schedule was initiated at diagnosis [61,62]. Whether or not these data justify the de-escalation of AR based on response depth still has not been tested prospectively. We know from the Magnolia trial that 24% of all cases of PBD were indeed observed in patients who had obtained CR in their latest line of treatment [63]. Furthermore, it has been demonstrated that stopping ZA after 12 months of treatment results in a shorter suppression of bone resorption, reflected by lower levels of bone resorption markers, compared to 24 months of treatment [64]. It thus seems that 48 months of treatment with AR is superior to only 24 months of treatment. Whether dosing can be reduced to every 12 weeks, or if patients with a certain depth of response may tolerate less treatment, remains to be tested prospectively. A future alternative approach to determine which patients can safely descale or pause AR treatment could perhaps be taking measurements of circulating microRNA. MicroRNAs have recently been shown to play an important role in osteoblasto- as well as osteoclastogenesis [65]. In addition, the same microRNAs are able to accurately predict the presence of osteolytic bone disease in newly diagnosed multiple myeloma [66].

Importantly, however, discontinuation of ZA and Dmab have very different effects on bone remodeling. Discontinuation of ZA results in a gradual declining effect, while discontinuation of Dmab may result in a devastating resorptive rebound phenomenon, probably due to an underlying upregulation of RANKL. Studies including patients with other diseases than MM have shown that terminating treatment with Dmab, or even a brief temporary discontinuation, may result in severe bone resorptive rebound, leading to an increased risk of multiple vertebral fractures [67,68,69,70]. A direct transition from Dmab to ZA has shown to diminish, but not prevent, this rebound phenomenon [71], and this approach may not be viable in patients on Dmab with reduced renal function. A practical approach in these patients could be to not discontinue treatment with Dmab but only reduce the intensity to every 12 weeks. Another proposed way to counter this problem is a combined short-term regimen with teriparatide and Dmab [72]. The Federal Drug Administration (FDA), however, has placed a black box warning on teriparatide for patients with skeletal malignancies as it increases the risk of osteosarcoma in rats. 

If AR treatment is completely discontinued, we know from the Azabache trial that it should be re-initiated at biochemical progression [73]. 

## 5. Vertobroplasty and Kyphoplasty

Vertebral compression fractures are a common complication of myeloma bone disease, resulting in severe pain and functional impairment. Surgical interventions, such as vertebroplasty and kyphoplasty, have emerged as effective treatment options for pain relief and the restoration of vertebral height in these patients [74,75]. Vertebral augmentation, vertebroplasty, and kyphoplasty are minimally invasive procedures performed under fluoroscopic guidance. Vertebroplasty involves the percutaneous injection of polymethylmethacrylate (PMMA) into the fractured vertebral body, providing stabilization. Kyphoplasty includes an additional step of balloon inflation to restore vertebral height before PMMA injection. These procedures are typically performed on an outpatient basis under local anesthesia with light sedation. The indications for vertebroplasty and kyphoplasty in patients with MM with vertebral compression fractures include symptomatic fractures and severe pain that is unresponsive to conservative management [75]. The protection of sagittal balance has emerged as an important indication, with increasing long-term survival rates in the MM population [75].

**Figure 2:**
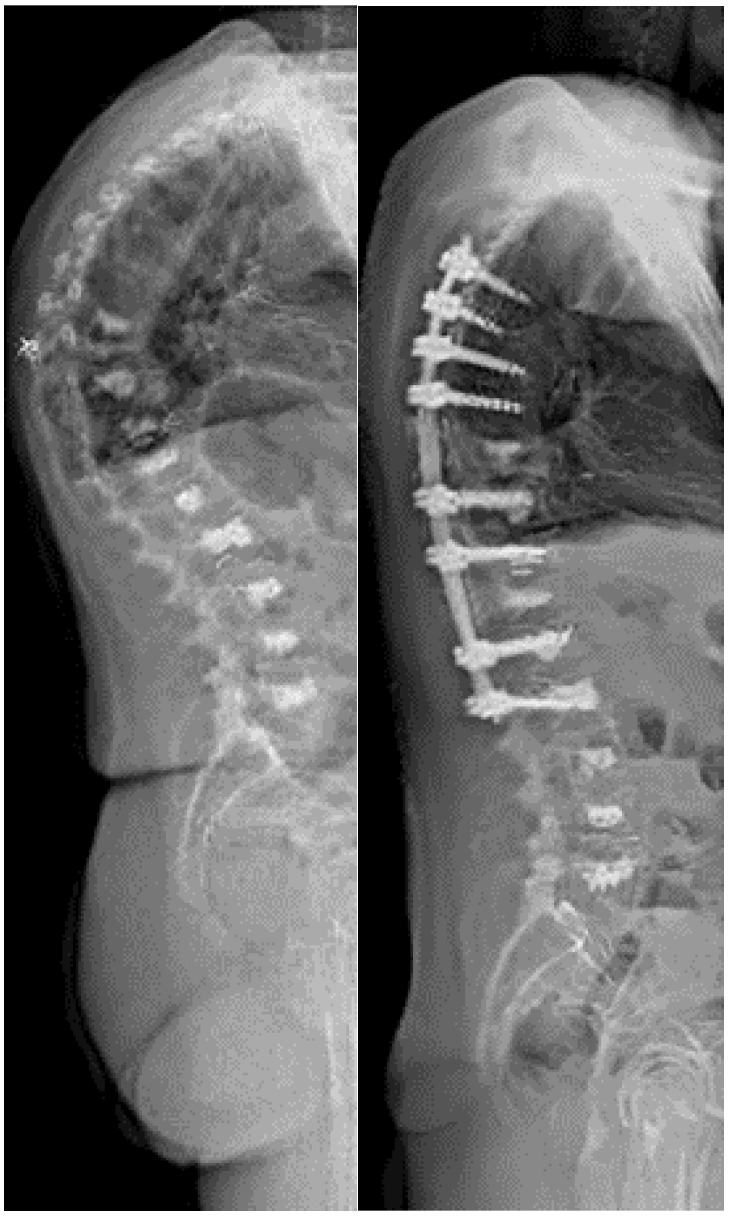
Lateral standing X-ray images before (**left**) and after (**right**) wedge osteotomy in a patient with MM with multiple vertebral lesions treated with vertebroplasty. Note the correction of the sagittal profile.

Several studies have reported favorable clinical outcomes following vertebral augmentation procedures in patients with MM [74,76,77]. Significant pain relief, improved mobility, and the restoration of vertebral height have been observed [74,76,78]. These interventions have shown a reduction in pain scores, decreased analgesic requirements, and improvements in quality of life [74,76,78]. Moreover, they are associated with low complication rates and minimal perioperative morbidity [74,79,80,81]. A Danish national clinical guideline for the treatment of malignant lesions with percutaneous vertebroplasty, published in 2020, includes a weak recommendation for the procedure [82]. However, the number of high-quality studies to uncover the full impact of the procedures is still low. Presently, a single-blinded, randomized clinical trial is being conducted to compare the outcomes of standard care alone versus standard care supplemented with vertebroplasty (clinicaltrials.gov: NCT04533217, accessed on 1 November 2023) [83]. Wedge osteotomy can be considered in cases where severe kyphotic deformity affects the patient’s activities of daily living. It allows for the reestablishment of sagittal balance and correction of deformity (Figure 2).

## 6. Targeting the Microenviroment

**Figure 3:**
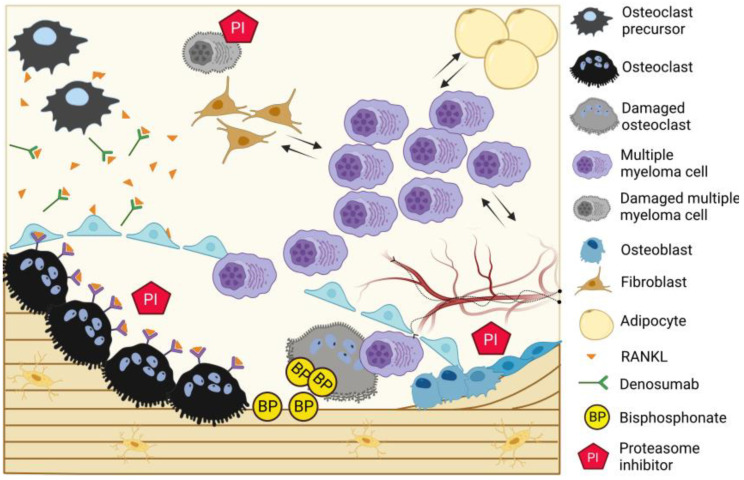
Current therapies in myeloma bone disease. MM cells interact with their surrounding bone marrow microenvironment, including vasculature/innervation, SSPCs, and adipocytes, which, in turn, promote MM cell survival and proliferation. Upon disruption of the bone remodeling compartment (BRC) canopy, MM cells enter into direct contact with osteoclasts, osteoblasts, and osteoprogenitor cells, uncoupling the bone resorption and formation and inducing osteolytic disease. Bisphosphonates bind the bone surface and promote osteoclast apoptosis and Dmab binds RANKL, thereby decreasing osteoclast differentiation. Ixazomib, a proteasome inhibitor, decreases osteoclast activity and simultaneously promotes longer bone formation events by osteoblasts, resulting in net bone gain.

Myeloma bone disease is characterized by the development of focal “punch-out” lesions, which are the result of a highly active bone resorption with an uncoupling of the subsequent bone formation. The uncoupled bone formation is likely a consequence of myeloma cell-induced disruption of the bone remodeling compartment (BRC) canopies (Figure 3), which reflect a bone marrow envelope that is lifted above remodeling sites [84]. The canopies/envelope consist of elongated osteoprogenitor cells that physically separate the bone surface cells, including remodeling events from the bone marrow cavity [10,85], considered to be a local reservoir of osteoprogenitor cells [84,86]. This local reservoir is critical for the transition from bone erosion to formation, requiring the recruitment of a critical density of osteoprogenitor cells to the eroded surfaces formed by bone-resorbing osteoclasts. It has previously been shown that the number of osteolytic lesions in patients with MM is directly correlated to the uncoupling of bone resorption and bone formation and that this uncoupling occurs primarily upon MM disruption of the canopies [10]. The mechanisms of canopy disruption are not fully understood, but it may be mediated by cancer-induced apoptosis of the canopy cells [11], as myeloma cells have been shown to induce apoptosis in osteoblastic cells via tumor necrosis factor-related apoptosis-inducing ligand (TRAIL) and Fas-Ligand (FasL) [87,88].

Upon gaining proximity to the bone surface cells, the MM cells upregulate osteoclast activity and differentiation along with osteoblast hypoactivity, altering the tightly coupled process of bone remodeling. The bidirectional effect of myeloma on bone cells is mediated by a myriad of osteoclast-activating factors, such as RANKL, macrophage inflammatory protein alpha (MIP-1 alpha), interleukin-1 (IL-1), interleukin-3 (IL-3), and tumor necrosis factor alpha (TNF-α) [89,90,91], and osteoblast-inhibiting factors like dickkopf WNT signaling pathway inhibitor 1 (DKK1), sclerostin, hepatocyte growth factor (HGF), interleukin-7 (IL-7), and TNF-α [92,93,94,95,96]. MM cells additionally express syndecan-1, which binds OPG, resulting in its endocytosis and degradation, further contributing to osteoclastogenesis [97]. In addition to the autocrine effect of myeloma cells on osteoclasts and osteoblasts, their modulation of the proximal bone marrow microenvironment also results in the further release of RANKL, macrophage colony-stimulating factor (MCSF), interleukin-6 (IL6), and TNF-α from bone marrow stromal cells [98,99,100] or osteoblast inhibitory factors like sclerostin from osteocytes [26,28,101,102,103], among others. Increased bone resorption leads to the release of growth factors from the bone matrix, which, in turn, promote cancer cell growth, leading to a reciprocal interaction known as the “vicious cycle”.

In addition to their direct and indirect effects on bone cells, myeloma cells modify other bone marrow microenvironment components that consequently support their engraftment, growth, and survival. Co-culture experiments demonstrate that MM cells inhibit adipocyte differentiation, promote adipocyte lipolysis, and uptake fatty acids from proximal adipocytes [104,105]. In turn, myeloma-modified adipocytes support cancer growth and survival, demonstrating a shift towards a pro-tumorigenic microenvironment upon cancer cell colonization. 

Skeletal stem and progenitor cells (SSPCs, known as mesenchymal stem cells) also play a crucial role in MBD and are distinctly altered by the presence of MM cells in both a paracrine and an autocrine fashion. SSPCs reversely alter MM cell phenotype and function by the secretion of micro-vesicles and cytokines that stimulate MM cell proliferation and migration [106,107,108], contributing to the establishment of a pro-tumorigenic microenvironment. In turn, SSPCs differentiation capacity is hampered in MM [109,110,111], resulting in osteoblastopenia [112] and an accumulation of SSPCs in the bone marrow [113]. Furthermore, SSPC gene expression is largely altered by MM, as demonstrated by in vitro co-culture studies of SSPCs with MM cell lines [114,115]. Even after ex vivo expansion of SSPCs without MM cells, the gene expression profiles of SSPCs from patients with MM are distinguishable from SSPCs from patients with premalignant monoclonal gammopathy of undetermined significance (MGUS; an asymptomatic condition that precedes MM) and healthy donors [114,116,117,118,119]. Deregulated transcriptional pathways include cell cycle regulation, osteoblast maturation, MM cell survival factors, and immune-modulating factors [117,119]. The deregulation of gene expression in SSPCs persists after anti-myeloma therapy [113,118,119], even in patients who are negative for measurable residual disease [119], suggesting a permanent modulation of the cells. This is supported by findings of genomic alterations [120] and epigenetic modifications of the SSPC genomes in MM, dependent on disease stage and inducible by MM cell co-culture [121]. While recent years’ advances in anti-myeloma therapy have improved survival in patients with MM tremendously [122], there has been no revolution in the treatment of myeloma bone disease, which still relies mainly on AR therapies.

Novel treatments targeting alterations of SSPCs could suppress the pro-tumorigenic bone marrow microenvironment while stimulating the differentiation of osteoblasts with bone-forming capacity and even anti-myeloma effects [123,124]. Proteasome inhibitors have been shown to exert some of their therapeutic effects, not only by their anti-tumor effect on myeloma cells but also through the off-target inhibition of the nuclear factor-kB (NF-kB) signaling pathway, which results in decreased RANKL-mediated osteoclastic differentiation [125]. Indeed, Bortezomib increases serum bone formation markers and decreases markers of bone resorption in clinical studies [126,127]. Another proteasome inhibitor, carfilzomib has been shown to promote increased trabecular bone volume in a mouse model of MM [128]. Furthermore, in a clinical trial with humans, carfilzomib has been shown to increase the bone formation markers osteocalcin and procollagen type I N-propeptide, independently of myeloma response to treatment [129]. 

The proteasome inhibitors’ improvement of the skeletal compartment is, however, overshadowed by problematic adverse toxicities, such as neuropathy and cardiac toxicity [130,131,132], and by the development of resistance in MM cells. Ixazomib, the first oral proteasome inhibitor, has proven to be more tolerable in clinical trials. In addition to its anti-tumor effect [133], it also improves osteoblast differentiation while inhibiting osteoclast differentiation in in vitro studies [134,135]. The effect of ixazomib on myeloma bone disease is currently being investigated in a clinical trial on patients with MM in remission (clinaltrials.gov NCT04028115, accessed on 1 November 2023). Recently published preliminary results from this trial [136] revealed a drug-mediated increase in trabecular bone volume mediated by decreased osteoclast activity and longer bone formation events in bone biopsies taken after just 3 months of treatment [136]. Daratumumab, a CD38 antibody that is extensively used to treat MM, has also demonstrated positive effects on bone formation. Whether this is due to a direct effect on the osteoblasts or through an indirect effect on the myeloma cells needs further exploration [137]. A quick overview of possible bone-targeting therapies can be obtained from Table 1. 

Importantly, the sum of the described MM-induced alterations to the bone marrow microenvironment drives the production of anti-apoptotic cytokines and suppresses anti-tumor responses, resulting in an environment supporting MM cells’ growth, survival, and resistance to therapy—termed “the MM niche”. This niche plays a pivotal role in the induction of MM dormancy [142,143], a state of quiescent growth arrest that allows MM cells to become unavailable to anti-myeloma therapy. Here, osteoblastic cells may control dormancy induction, while osteoclastic bone remodeling may promote dormancy escape and consequent disease relapse [144]. These residual MM cells accumulate mutations over time, driving later disease relapses and resulting in the incurable disease that is MM; thus, targeting myeloma bone disease may ultimately pose an effective avenue towards disease cure. 

## 7. Conclusions and Future Perspectives

Myeloma bone disease remains a challenging condition to manage. Vertebral compression fractures are painful and immobilizing and thereby are potentially dangerous. Surgical interventions with a focus on pain relief and restoring the sagittal balance indicate a very low perioperative risk versus a great therapeutic value, but the full therapeutic impact still needs to be investigated further. AR treatment is necessary for patients with MM, but, for a small number, it can bring debilitating adverse events, such as MRONJ, that is difficult to manage. AR therapy regimes could benefit from prolonging treatment periods from two to four years in order to reduce the risk of PDB. But, since the incidence of MRONJ rises with the cumulative dosage of antiresorptive medication, the treating physician might consider reducing the dosing frequency to every 3rd month for patients who achieve VGPR or better after their anti-myeloma therapy.

In the treatment of MRONJ, conservative treatment was previously the preferred option, but, now, surgery is recommended and is successful in 60–85% of patients. Overall, the treatment protocols lack high-grade evidence, and several adjuvating therapies are being investigated. No consensus has been reached on drug holidays and whether these are beneficial in preventing MRONJ, and the preferable and most effective measure seems to be the prevention of MRONJ by preventing infection and inflammation in the bone by regular dental examinations.

A major breakthrough in myeloma bone disease treatment is still awaited, and research should continue to investigate bone anabolic treatments. Romosozumab, the anti-sclerostin antibody approved in treatments for osteoporosis, could be relevant in the treatment of myeloma bone disease. Further, proteasome inhibitors stimulating the stromal cells in the bone marrow microenvironment show promise in inducing bone healing and also potentially diminishing the risk of myeloma progression. Ixazomib, the first oral proteasome inhibitor, is currently being investigated in this respect, and preliminary data are promising.

## Figures and Tables

**Table 1:** Major or novel bone targeting therapies and their mechanism of action.

Drug	Target	Pathway	Mechanism of Action	Reference
Zoledronic Acid (bisphosphonate)	Osteoclasts	Mevalonate pathway	Inhibits bone resorption	[138]
Denosumab (monoclonal antibody)	RANK	RANK/RANKL pathway	Inhibits bone resorption	[12]
Daratumumab (monoclonal antibody)	CD38	Antibody-dependent cellular cytotoxicity (ADCC) Complement-dependent cytotoxicity (CDC) Direct apoptosis	Signaling events, receptor-mediated adhesion, regulation of migration	[139]
Romosozumab (monoclonal antibody)	Sclerostin	Wnt-signaling pathway	Inhibits osteoclastogenesis and perhaps stimulates bone formation	[140]
Ixazomib (proteasome inhibitor)	20S proteasome (proteasome subunit beta type-5)	Ubiquitin-proteasome pathway	Inhibition of NF-κB signaling Cell cycle arrest Cell apoptosis Stimulates osteoblast differentiation Inhibits osteoclast differentiation	[141]

## References

[1] Zhang N., Wu J., Wang Q., Liang Y., Li X., Chen G., Ma L., Liu X., Zhou F. (2023). Global burden of hematologic malignancies and evolution patterns over the past 30 years. Blood Cancer J..

[2] Sant M., Allemani C., Tereanu C., De Angelis R., Capocaccia R., Visser O., Marcos-Gragera R., Maynadié M., Simonetti A., Lutz J.M. (2010). Incidence of hematologic malignancies in Europe by morphologic subtype: Results of the HAEMACARE project. Blood.

[3] Kyle R.A., Gertz M.A., Witzig T.E., Lust J.A., Lacy M.Q., Dispenzieri A., Fonseca R., Rajkumar S.V., Offord J.R., Larson D.R. (2003). Review of 1027 patients with newly diagnosed multiple myeloma. Mayo Clin Proc..

[4] Terpos E., Berenson J., Cook R.J., Lipton A., Coleman R.E. (2010). Prognostic variables for survival and skeletal complications in patients with multiple myeloma osteolytic bone disease. Leukemia.

[5] Royle K., Gregory W.M., Cairns D.A., Bell S.E., Cook G., Owen R.G., Drayson M.T., Davies F.E., Jackson G.H., Morgan G.J. (2018). Quality of life during and following sequential treatment of previously untreated patients with multiple myeloma: Findings of the Medical Research Council Myeloma IX randomised study. Br. J. Haematol..

[6] Nielsen L.K., Larsen R.F., Jarlbaek L., Möller S., Jespersen E. (2021). Health-related quality of life in patients with multiple myeloma participating in a multidisciplinary rehabilitation program. Ann. Hematol..

[7] Horsboel T.A., Nielsen C.V., Andersen N.T., Nielsen B., de Thurah A. (2014). Risk of disability pension for patients diagnosed with haematological malignancies: A register-based cohort study. Acta Oncol..

[8] Sonmez M., Akagun T., Topbas M., Cobanoglu U., Sonmez B., Yilmaz M., Ovali E., Omay S.B. (2008). Effect of pathologic fractures on survival in multiple myeloma patients: A case control study. J. Exp. Clin. Cancer Res..

[9] Capp J.-P., Bataille R. (2023). The Ins and Outs of Endosteal Niche Disruption in the Bone Marrow: Relevance for Myeloma Oncogenesis. Biology.

[10] Andersen T.L., Sondergaard T.E., Skorzynska K.E., Dagnaes-Hansen F., Plesner T.L., Hauge E.M., Plesner T., Delaisse J.M. (2009). A physical mechanism for coupling bone resorption and formation in adult human bone. Am. J. Pathol..

[11] Andersen T.L., Søe K., Sondergaard T.E., Plesner T., Delaisse J.M. (2010). Myeloma cell-induced disruption of bone remodelling compartments leads to osteolytic lesions and generation of osteoclast-myeloma hybrid cells. Br. J. Haematol..

[12] Casimiro S., Vilhais G., Gomes I., Costa L. (2021). The Roadmap of RANKL/RANK Pathway in Cancer. Cells.

[13] Boyce B.F., Xing L. (2007). Biology of RANK, RANKL, and osteoprotegerin. Arthritis Res. Ther..

[14] Depil S., Mathiot C., Leleu X., Moreau A.S., Faucompre J.-L., Hennache B., Bauters F., Bataille R., Facon T. (2005). Evaluation and prognostic value of serum osteoprotegerin in multiple myeloma. Br. J. Haematol..

[15] Terpos E., Morgan G., Dimopoulos M.A., Drake M.T., Lentzsch S., Raje N., Sezer O., García-Sanz R., Shimizu K., Turesson I. (2013). International Myeloma Working Group recommendations for the treatment of multiple myeloma-related bone disease. J. Clin. Oncol..

[16] Drake M.T., Clarke B.L., Khosla S. (2008). Bisphosphonates: Mechanism of action and role in clinical practice. Mayo Clin. Proc..

[17] Terpos E., Zamagni E., Lentzsch S., Drake M.T., García-Sanz R., Abildgaard N., Ntanasis-Stathopoulos I., Schjesvold F., de la Rubia J., Kyriakou C. (2021). Treatment of multiple myeloma-related bone disease: Recommendations from the Bone Working Group of the International Myeloma Working Group. Lancet Oncol..

[18] Stein E., Dash A., Bucovsky M., Agarwal S., Fu J., Lentzsch S., Shane E. (2019). Disrupted radial and tibial microarchitecture in patients with monoclonal gammopathy of undetermined significance. Osteoporos. Int..

[19] Rosen L.S., Gordon D., Kaminski M., Howell A., Belch A., Mackey J., Apffelstaedt J., Hussein M., Coleman R.E., Reitsma D.J. (2001). Zoledronic acid versus pamidronate in the treatment of skeletal metastases in patients with breast cancer or osteolytic lesions of multiple myeloma: A phase III, double-blind, comparative trial. Cancer J..

[20] Sanfilippo K.M., Gage B., Luo S., Weilbaecher K., Tomasson M., Vij R., Colditz G., Carson K. (2015). Comparative effectiveness on survival of zoledronic acid versus pamidronate in multiple myeloma. Leuk. Lymphoma.

[21] Mhaskar R., Kumar A., Miladinovic B., Djulbegovic B. (2017). Bisphosphonates in multiple myeloma: An updated network meta-analysis. Cochrane Database Syst. Rev..

[22] Rosen L.S., Gordon D., Kaminski M., Howell A., Belch A., Mackey J., Apffelstaedt J., Hussein M.A., Coleman R.E., Reitsma D.J. (2003). Long-term efficacy and safety of zoledronic acid compared with pamidronate disodium in the treatment of skeletal complications in patients with advanced multiple myeloma or breast carcinoma: A randomized, double-blind, multicenter, comparative trial. Cancer.

[23] Major P., Lortholary A., Hon J., Abdi E., Mills G., Menssen H.D., Yunus F., Bell R., Body J., Quebe-Fehling E. (2001). Zoledronic acid is superior to pamidronate in the treatment of hypercalcemia of malignancy: A pooled analysis of two randomized, controlled clinical trials. J. Clin. Oncol..

[24] Raje N., Terpos E., Willenbacher W., Shimizu K., García-Sanz R., Durie B., Legieć W., Krejčí M., Laribi K., Zhu L. (2018). Denosumab versus zoledronic acid in bone disease treatment of newly diagnosed multiple myeloma: An international, double-blind, double-dummy, randomised, controlled, phase 3 study. Lancet Oncol..

[25] Terpos E., Raje N., Croucher P., Garcia-Sanz R., Leleu X., Pasteiner W., Wang Y., Glennane A., Canon J., Pawlyn C. (2021). Denosumab compared with zoledronic acid on PFS in multiple myeloma: Exploratory results of an international phase 3 study. Blood Adv..

[26] Delgado-Calle J., Anderson J., Cregor M.D., Hiasa M., Chirgwin J.M., Carlesso N., Yoneda T., Mohammad K.S., Plotkin L.I., Roodman G.D. (2016). Bidirectional Notch Signaling and Osteocyte-Derived Factors in the Bone Marrow Microenvironment Promote Tumor Cell Proliferation and Bone Destruction in Multiple Myeloma. Cancer Res..

[27] McDonald M.M., Reagan M.R., Youlten S.E., Mohanty S.T., Seckinger A., Terry R.L., Pettitt J.A., Simic M.K., Cheng T.L., Morse A. (2017). Inhibiting the osteocyte-specific protein sclerostin increases bone mass and fracture resistance in multiple myeloma. Blood.

[28] Delgado-Calle J., Anderson J., Cregor M.D., Condon K.W., A Kuhstoss S., Plotkin L.I., Bellido T., Roodman G.D. (2017). Genetic deletion of Sost or pharmacological inhibition of sclerostin prevent multiple myeloma-induced bone disease without affecting tumor growth. Leukemia.

[29] Gadgaard N.R., Olesen T.B., Svane H.M.L., Heide-Jørgensen U., Nørholt S.E., Ehrenstein V. (2022). Osteonecrosis of the jaw among cancer patients in Denmark: Risk and prognosis. Int. J. Oral. Maxillofac. Surg..

[30] Morgan G.J., E Davies F., Gregory W.M., Cocks K., E Bell S., Szubert A.J., Navarro-Coy N., Drayson M.T., Owen R.G., Feyler S. (2010). First-line treatment with zoledronic acid as compared with clodronic acid in multiple myeloma (MRC Myeloma IX): A randomised controlled trial. Lancet.

[31] Hoff A.O., Toth B.B., Altundag K., Johnson M.M., Warneke C.L., Hu M., Nooka A., Sayegh G., Guarneri V., Desrouleaux K. (2008). Frequency and risk factors associated with osteonecrosis of the jaw in cancer patients treated with intravenous bisphosphonates. J. Bone Miner. Res..

[32] Zhang C., Shen G., Li H., Xin Y., Shi M., Zheng Y., Wang M., Liu Z., Zhao Y., Zhao F. (2023). Incidence rate of osteonecrosis of jaw after cancer treated with bisphosphonates and denosumab: A systematic review and meta-analysis. Spec. Care Dent..

[33] Schiodt M., Otto S., Fedele S., Bedogni A., Nicolatou-Galitis O., Guggenberger R., Herlofson B.B., Ristow O., Kofod T. (2019). Workshop of European task force on medication-related osteonecrosis of the jaw-Current challenges. Oral. Dis..

[34] Otto S., Tröltzsch M., Jambrovic V., Panya S., Probst F., Ristow O., Ehrenfeld M., Pautke C. (2015). Tooth extraction in patients receiving oral or intravenous bisphosphonate administration: A trigger for BRONJ development?. J. Craniomaxillofac. Surg..

[35] Panya S., Fliefel R., Probst F., Tröltzsch M., Ehrenfeld M., Schubert S., Otto S. (2017). Role of microbiological culture and polymerase chain reaction (PCR) of actinomyces in medication-related osteonecrosis of the jaw (MRONJ). J. Craniomaxillofac. Surg..

[36] Otto S., Aljohani S., Fliefel R., Ecke S., Ristow O., Burian E., Troeltzsch M., Pautke C., Ehrenfeld M. (2021). Infection as an Important Factor in Medication-Related Osteonecrosis of the Jaw (MRONJ). Medicina.

[37] Beth-Tasdogan N.H., Mayer B., Hussein H., Zolk O., Peter J.U. (2022). Interventions for managing medication-related osteonecrosis of the jaw. Cochrane Database Syst. Rev..

[38] Montefusco V., Gay F., Spina F., Miceli R., Maniezzo M., Teresa Ambrosini M., Farina L., Piva S., Palumbo A., Boccadoro M. (2008). Antibiotic prophylaxis before dental procedures may reduce the incidence of osteonecrosis of the jaw in patients with multiple myeloma treated with bisphosphonates. Leuk. Lymphoma.

[39] Schubert M., Klatte I., Linek W., Müller B., Döring K., Eckelt U., Hemprich A., Berger U., Hendricks J. (2012). The saxon bisphosphonate register—Therapy and prevention of bisphosphonate-related osteonecrosis of the jaws. Oral. Oncol..

[40] Andersen S.W.M., Jensen S.S., Schiodt M. (2021). Apical surgery in cancer patients receiving high-dose antiresorptive medication-a retrospective clinical study with a mean follow-up of 13 months. Oral. Maxillofac. Surg..

[41] Mücke T., Deppe H., Hein J., Wolff K.-D., Mitchell D.A., Kesting M.R., Retz M., Gschwend J.E., Thalgott M. (2016). Prevention of bisphosphonate-related osteonecrosis of the jaws in patients with prostate cancer treated with zoledronic acid—A prospective study over 6 years. J. Craniomaxillofac. Surg..

[42] Aboalela A.A., Farook F.F., Alqahtani A.S., A Almousa M., Alanazi R.T., Almohammadi D.S. (2022). The Effect of Antiresorptive Drug Holidays on Medication-Related Osteonecrosis of the Jaw: A Systematic Review and Meta-Analysis. Cureus.

[43] Ottesen C., Schiodt M., Jensen S.S., Kofod T., Gotfredsen K. (2022). Tooth extractions in patients with cancer receiving high-dose antiresorptive medication: A randomized clinical feasibility trial of drug holiday versus drug continuation. Oral. Surg. Oral. Med. Oral. Pathol. Oral. Radiol..

[44] Ruggiero S.L., Dodson T.B., Aghaloo T., Carlson E.R., Ward B.B., Kademani D. (2022). American Association of Oral and Maxillofacial Surgeons’ Position Paper on Medication-Related Osteonecrosis of the Jaws-2022 Update. J. Oral. Maxillofac. Surg..

[45] Ristow O., Rückschloß T., Müller M., Berger M., Kargus S., Pautke C., Engel M., Hoffmann J., Freudlsperger C. (2019). Is the conservative non-surgical management of medication-related osteonecrosis of the jaw an appropriate treatment option for early stages? A long-term single-center cohort study. J. Craniomaxillofac. Surg..

[46] Andersen S.W.M., Mogensen D.G., Schioedt M., Kofod T. (2023). Surgical treatment of 61 consecutive patients with maxillary stage 3 medication-related osteonecrosis of the jaws using a pedicled buccal fat pad. Oral. Maxillofac. Surg..

[47] Govaerts D., Piccart F., Ockerman A., Coropciuc R., Politis C., Jacobs R. (2020). Adjuvant therapies for MRONJ: A systematic review. Bone.

[48] Sim I.-W., Borromeo G.L., Tsao C., Hardiman R., Hofman M.S., Hjelle C.P., Siddique M., Cook G.J.R., Seymour J.F., Ebeling P.R. (2020). Teriparatide Promotes Bone Healing in Medication-Related Osteonecrosis of the Jaw: A Placebo-Controlled, Randomized Trial. J. Clin. Oncol..

[49] Ohbayashi Y., Iwasaki A., Nakai F., Mashiba T., Miyake M. (2020). A comparative effectiveness pilot study of teriparatide for medication-related osteonecrosis of the jaw: Daily versus weekly administration. Osteoporos. Int..

[50] Giudice A., Barone S., Giudice C., Bennardo F., Fortunato L. (2018). Can platelet-rich fibrin improve healing after surgical treatment of medication-related osteonecrosis of the jaw? A pilot study. Oral. Surg. Oral. Med. Oral. Pathol. Oral. Radiol..

[51] Park J.H., Kim J.W., Kim S.J. (2017). Does the Addition of Bone Morphogenetic Protein 2 to Platelet-Rich Fibrin Improve Healing After Treatment for Medication-Related Osteonecrosis of the Jaw?. J. Oral Maxillofac. Surg..

[52] Ristow O., Otto S., Geiß C., Kehl V., Berger M., Troeltzsch M., Koerdt S., Hohlweg-Majert B., Freudlsperger C., Pautke C. (2017). Comparison of auto-fluorescence and tetracycline fluorescence for guided bone surgery of medication-related osteonecrosis of the jaw: A randomized controlled feasibility study. Int. J. Oral. Maxillofac. Surg..

[53] Giudice A., Bennardo F., Barone S., Antonelli A., Figliuzzi M.M., Fortunato L. (2018). Can Autofluorescence Guide Surgeons in the Treatment of Medication-Related Osteonecrosis of the Jaw? A Prospective Feasibility Study. J. Oral. Maxillofac. Surg..

[54] Black D.M., Geiger E.J., Eastell R., Vittinghoff E., Li B.H., Ryan D.S., Dell R.M., Adams A.L. (2020). Atypical Femur Fracture Risk versus Fragility Fracture Prevention with Bisphosphonates. N. Engl. J. Med..

[55] Ferreira P., Bates P., Daoub A., Dass D. (2023). Is bisphosphonate use a risk factor for atypical periprosthetic/peri-implant fractures?—A metanalysis of retrospective cohort studies and systematic review of the current evidence. Orthop. Traumatol. Surg. Res..

[56] Berenson J.R., Lichtenstein A., Porter L., Dimopoulos M.A., Bordoni R., George S., Lipton A., Keller A., Ballester O., Kovacs M.J. (1996). Efficacy of pamidronate in reducing skeletal events in patients with advanced multiple myeloma. Myeloma Aredia Study Group. N. Engl. J. Med..

[57] Avilès A., Nambo M.-J., Huerta-Guzmàn J., Cleto S., Neri N. (2017). Prolonged Use of Zoledronic Acid (4 Years) Did Not Improve Outcome in Multiple Myeloma Patients. Clin. Lymphoma Myeloma Leuk..

[58] Gundesen M., Schjesvold F., Vangsted A.J., Helleberg C., Haukås E., Silkjær T., Teodorescu E., Jensen B., Slordahl T., Asmussen J. (2023). OA-10 Four years treatment with zoledronic acid is superior to two years in protection against progressive bone disease in multiple myeloma. Clin. Lymphoma Myeloma Leuk..

[59] Larocca A., Child J.A., Cook G., Jackson G.H., Russell N., Szubert A., Gregory W.M., Brioli A., Owen R.G., Drayson M.T. (2013). The impact of response on bone-directed therapy in patients with multiple myeloma. Blood.

[60] Gavriatopoulou M., Terpos E., Ntanasis-Stathopoulos I., Malandrakis P., Eleutherakis-Papaiakovou E., Papatheodorou A., Kanellias N., Migkou M., Fotiou D., Dialoupi I. (2020). Consolidation with carfilzomib, lenalidomide, and dexamethasone (KRd) following ASCT results in high rates of minimal residual disease negativity and improves bone metabolism, in the absence of bisphosphonates, among newly diagnosed patients with multiple myeloma. Blood Cancer J..

[61] Himelstein A.L., Foster J.C., Khatcheressian J.L., Roberts J.D., Seisler D.K., Novotny P.J., Qin R., Go R.S., Grubbs S.S., O’Connor T. (2017). Effect of Longer-Interval vs Standard Dosing of Zoledronic Acid on Skeletal Events in Patients With Bone Metastases: A Randomized Clinical Trial. JAMA.

[62] Hortobagyi G.N., Van Poznak C., Harker W.G., Gradishar W.J., Chew H., Dakhil S.R., Haley B.B., Sauter N., Mohanlal R., Zheng M. (2017). Continued Treatment Effect of Zoledronic Acid Dosing Every 12 vs 4 Weeks in Women With Breast Cancer Metastatic to Bone: The OPTIMIZE-2 Randomized Clinical Trial. JAMA Oncol..

[63] Gundesen M.T., Asmussen J.T., Schjesvold F., Vangsted A.J., Helleberg C., Haukås E., Silkjær T., Teodorescu E.M., Jensen B.A., Slørdahl T.S. (2023). Potential value of pre-planned imaging of bone disease in multiple myeloma. Blood Cancer J..

[64] Lund T., Abildgaard N., Delaisse J.-M., Plesner T. (2010). Effect of withdrawal of zoledronic acid treatment on bone remodelling markers in multiple myeloma. Br. J. Haematol..

[65] Papanota A.-M., Karousi P., Kontos C.K., Ntanasis-Stathopoulos I., Scorilas A., Terpos E. (2021). Multiple Myeloma Bone Disease: Implication of MicroRNAs in Its Molecular Background. Int. J. Mol. Sci..

[66] Papanota A.-M., Tsiakanikas P., Kontos C.K., Malandrakis P., Liacos C.-I., Ntanasis-Stathopoulos I., Kanellias N., Gavriatopoulou M., Kastritis E., Avgeris M. (2021). A Molecular Signature of Circulating MicroRNA Can Predict Osteolytic Bone Disease in Multiple Myeloma. Cancers.

[67] Drejer L.A., El-Masri B.M., Ejersted C., Andreasen C.M., Thomsen L.K., Thomsen J.S., Andersen T.L., Hansen S. (2023). Trabecular bone deterioration in a postmenopausal female suffering multiple spontaneous vertebral fractures due to a delayed denosumab injection—A post-treatment re-initiation bone biopsy-based case study. Bone Rep..

[68] Cummings S.R., Ferrari S., Eastell R., Gilchrist N., Jensen J.B., McClung M., Roux C., Törring O., Valter I., Wang A.T. (2018). Vertebral Fractures After Discontinuation of Denosumab: A Post Hoc Analysis of the Randomized Placebo-Controlled FREEDOM Trial and Its Extension. J. Bone Miner. Res..

[69] Lyu H., Yoshida K., Zhao S.S., Wei J., Zeng C., Tedeschi S.K., Leder B.Z., Lei G., Tang P., Solomon D.H. (2020). Delayed Denosumab Injections and Fracture Risk Among Patients With Osteoporosis: A Population-Based Cohort Study. Ann. Intern. Med..

[70] Burckhardt P., Faouzi M., Buclin T., Lamy O. (2021). Fractures After Denosumab Discontinuation: A Retrospective Study of 797 Cases. J. Bone Miner. Res..

[71] Sølling A.S., Harsløf T., Langdahl B. (2020). Treatment with Zoledronate Subsequent to Denosumab in Osteoporosis: A Randomized Trial. J. Bone Miner. Res..

[72] Ramchand S.K., David N.L., Lee H., Eastell R., Tsai J.N., Leder B.Z. (2021). Efficacy of Zoledronic Acid in Maintaining Areal and Volumetric Bone Density After Combined Denosumab and Teriparatide Administration: DATA-HD Study Extension. J. Bone Miner. Res..

[73] García-Sanz R., Oriol A., Moreno M.J., de la Rubia J., Payer A.R., Hernández M.T., Palomera L., Teruel A.I., Blanchard M.J., Gironella M. (2015). Zoledronic acid as compared with observation in multiple myeloma patients at biochemical relapse: Results of the randomized AZABACHE Spanish trial. Haematologica.

[74] Dudeney S., Lieberman I., Reinhardt M.-K., Hussein M. (2002). Kyphoplasty in the treatment of osteolytic vertebral compression fractures as a result of multiple myeloma. J. Clin. Oncol..

[75] Kyriakou C., Molloy S., Vrionis F., Alberico R., Bastian L., Zonder J.A., Giralt S., Raje N., Kyle R.A., Roodman D.G.D. (2019). The role of cement augmentation with percutaneous vertebroplasty and balloon kyphoplasty for the treatment of vertebral compression fractures in multiple myeloma: A consensus statement from the International Myeloma Working Group (IMWG). Blood Cancer J..

[76] Malhotra K., Butler J.S., Yu H.M., Selvadurai S., D’sa S., Rabin N., Kyriakou C., Yong K., Molloy S. (2016). Spinal disease in myeloma: Cohort analysis at a specialist spinal surgery centre indicates benefit of early surgical augmentation or bracing. BMC Cancer.

[77] Audat Z.A., Hajyousef M.H., Fawareh M.D., Alawneh K.M., Odat M.A., Barbarawi M.M., Alomari A.A., Jahmani R.A., Khatatbeh M.A., Assmairan M.A. (2016). Comparison if the addition of multilevel vertebral augmentation to conventional therapy will improve the outcome of patients with multiple myeloma. Scoliosis Spinal Disord..

[78] Berenson J., Pflugmacher R., Jarzem P., Zonder J., Schechtman K., Tillman J.B., Bastian L., Ashraf T., Vrionis F. (2011). Balloon kyphoplasty versus non-surgical fracture management for treatment of painful vertebral body compression fractures in patients with cancer: A multicentre, randomised controlled trial. Lancet Oncol..

[79] Nas Ö.F., İnecikli M.F., Hacıkurt K., Büyükkaya R., Özkaya G., Özkalemkaş F., Ali R., Erdoğan C., Hakyemez B. (2016). Effectiveness of percutaneous vertebroplasty in patients with multiple myeloma having vertebral pain. Diagn. Interv. Radiol..

[80] Klazen C.A., Lohle P.N., de Vries J., Jansen F.H., Tielbeek A.V., Blonk M.C., Venmans A., van Rooij W.J., Schoemaker M.C., Juttmann J.R. (2010). Vertebroplasty versus conservative treatment in acute osteoporotic vertebral compression fractures (Vertos II): An open-label randomised trial. Lancet.

[81] Pflugmacher R., Taylor R., Agarwal A., Melcher I., Disch A., Haas N.P., Klostermann C. (2008). Balloon kyphoplasty in the treatment of metastatic disease of the spine: A 2-year prospective evaluation. Eur. Spine J..

[82] Rousing R., Kirkegaard A.O., Nielsen M., Holtved E., Sørensen L.H., Lund T., Olesen V., Andersen M. (2020). Percutaneous vertebroplasty as treatment of malignant vertebral lesions: A systematic review and GRADE evaluation resulting in a Danish national clinical guideline. Eur. Spine J..

[83] Wickstroem L.A., Carreon L., Lund T., Abildgaard N., Lorenzen M.D., Andersen M. (2021). Vertebroplasty in patients with multiple myeloma with vertebral compression fractures: Protocol for a single-blind randomised controlled trial. BMJ Open.

[84] Andersen T.L., Jensen P.R., Sikjaer T.T., Rejnmark L., Ejersted C., Delaisse J.M. (2023). A Critical Role of the Bone Marrow Envelope in Human Bone Remodeling. J. Bone Miner. Res..

[85] Hauge E.M., Qvesel D., Eriksen E.F., Mosekilde L., Melsen F. (2001). Cancellous bone remodeling occurs in specialized compartments lined by cells expressing osteoblastic markers. J. Bone Miner. Res..

[86] Kristensen H.B., Andersen T.L., Marcussen N., Rolighed L., Delaisse J.-M. (2014). Osteoblast recruitment routes in human cancellous bone remodeling. Am. J. Pathol..

[87] Silvestris F., Cafforio P., Tucci M., Grinello D., Dammacco F. (2003). Upregulation of osteoblast apoptosis by malignant plasma cells: A role in myeloma bone disease. Br. J. Haematol..

[88] Tinhofer I., Biedermann R., Krismer M., Crazzolara R., Greil R. (2006). A role of TRAIL in killing osteoblasts by myeloma cells. FASEB J..

[89] Lee J.W., Chung H.Y., Ehrlich L.A., Jelinek D.F., Callander N.S., Roodman G.D., Choi S.J. (2004). IL-3 expression by myeloma cells increases both osteoclast formation and growth of myeloma cells. Blood.

[90] Lichtenstein A., Berenson J., Norman D., Chang M.P., Carlile A. (1989). Production of cytokines by bone marrow cells obtained from patients with multiple myeloma. Blood.

[91] Giuliani N., Colla S., Sala R., Moroni M., Lazzaretti M., La Monica S., Bonomini S., Hojden M., Sammarelli G., Barillè S. (2002). Human myeloma cells stimulate the receptor activator of nuclear factor-kappa B ligand (RANKL) in T lymphocytes: A potential role in multiple myeloma bone disease. Blood.

[92] Politou M.C., Heath D.J., Rahemtulla A., Szydlo R., Anagnostopoulos A., Dimopoulos M.A., Croucher P.I., Terpos E. (2006). Serum concentrations of Dickkopf-1 protein are increased in patients with multiple myeloma and reduced after autologous stem cell transplantation. Int. J. Cancer.

[93] Brunetti G., Oranger A., Mori G., Specchia G., Rinaldi E., Curci P., Zallone A., Rizzi R., Grano M., Colucci S. (2011). Sclerostin is overexpressed by plasma cells from multiple myeloma patients. Ann. N. Y Acad. Sci..

[94] Kristensen I.B., Christensen J.H., Lyng M.B., Møller M.B., Pedersen L., Rasmussen L.M., Ditzel H.J., Abildgaard N. (2013). Hepatocyte growth factor pathway upregulation in the bone marrow microenvironment in multiple myeloma is associated with lytic bone disease. Br. J. Haematol..

[95] Giuliani N., Colla S., Morandi F., Lazzaretti M., Sala R., Bonomini S., Grano M., Colucci S., Svaldi M., Rizzoli V. (2005). Myeloma cells block RUNX2/CBFA1 activity in human bone marrow osteoblast progenitors and inhibit osteoblast formation and differentiation. Blood.

[96] Adamik J., Jin S., Sun Q., Zhang P., Weiss K.R., Anderson J.L., Silbermann R., Roodman G.D., Galson D.L. (2017). EZH2 or HDAC1 Inhibition Reverses Multiple Myeloma-Induced Epigenetic Suppression of Osteoblast Differentiation. Mol. Cancer Res..

[97] Standal T., Seidel C., Hjertner Ø., Plesner T., Sanderson R.D., Waage A., Borset M., Sundan A. (2002). Osteoprotegerin is bound, internalized, and degraded by multiple myeloma cells. Blood.

[98] Maiso P., Mogollón P., Ocio E.M., Garayoa M. (2021). Bone Marrow Mesenchymal Stromal Cells in Multiple Myeloma: Their Role as Active Contributors to Myeloma Progression. Cancers.

[99] Roux S., Meignin V., Quillard J., Meduri G., Guiochon-Mantel A., Fermand J.P., Milgrom E., Mariette X. (2002). RANK (receptor activator of nuclear factor-kappaB) and RANKL expression in multiple myeloma. Br. J. Haematol..

[100] Pearse R.N., Sordillo E.M., Yaccoby S., Wong B.R., Liau D.F., Colman N., Michaeli J., Epstein J., Choi Y. (2001). Multiple myeloma disrupts the TRANCE/osteoprotegerin cytokine axis to trigger bone destruction and promote tumor progression. Proc. Natl. Acad. Sci. USA.

[101] Liu H., He J., Bagheri-Yarmand R., Li Z., Liu R., Wang Z., Bach D.H., Huang Y.H., Lin P., Guise T.A. (2022). Osteocyte CIITA aggravates osteolytic bone lesions in myeloma. Nat. Commun..

[102] Delgado-Calle J., Bellido T., Roodman G.D. (2014). Role of osteocytes in multiple myeloma bone disease. Curr. Opin. Support. Palliat. Care.

[103] Calle J.D., Bellido T., Roodman G.D.D. (2013). Direct Cell-To-Cell Interactions Between Osteocytes and Multiple Myeloma (MM) Cells Upregulate Sost and Downregulate OPG Expression In Osteocytes: Evidence For Osteocytic Contributions To MM-Induced Bone Disease. Blood.

[104] Panaroni C., Fulzele K., Mori T., Siu K.T., Onyewadume C., Maebius A., Raje N. (2022). Multiple myeloma cells induce lipolysis in adipocytes and uptake fatty acids through fatty acid transporter proteins. Blood.

[105] Fairfield H., Costa S., Falank C., Farrell M., Murphy C.S., D’amico A., Driscoll H., Reagan M.R. (2020). Multiple Myeloma Cells Alter Adipogenesis, Increase Senescence-Related and Inflammatory Gene Transcript Expression, and Alter Metabolism in Preadipocytes. Front. Oncol..

[106] Dabbah M., Attar-Schneider O., Matalon S.T., Shefler I., Dolberg O.J., Lishner M., Drucker L. (2017). Microvesicles derived from normal and multiple myeloma bone marrow mesenchymal stem cells differentially modulate myeloma cells’ phenotype and translation initiation. Carcinogenesis.

[107] Ibraheem A., Attar-Schneider O., Dabbah M., Jarchowsky O.D., Matalon S.T., Lishner M., Drucker L. (2019). BM-MSCs-derived ECM modifies multiple myeloma phenotype and drug response in a source-dependent manner. Transl. Res..

[108] Dabbah M., Jarchowsky-Dolberg O., Attar-Schneider O., Matalon S.T., Pasmanik-Chor M., Drucker L., Lishner M. (2020). Multiple myeloma BM-MSCs increase the tumorigenicity of MM cells via transfer of VLA4-enriched microvesicles. Carcinogenesis.

[109] André T., Meuleman N., Stamatopoulos B., De Bruyn C., Pieters K., Bron D., Lagneaux L. (2013). Evidences of early senescence in multiple myeloma bone marrow mesenchymal stromal cells. PLoS ONE.

[110] Arnulf B., Lecourt S., Soulier J., Ternaux B., Lacassagne M.-N., Crinquette A., Dessoly J., Sciaini A.K., Benbunan M., Chomienne C. (2007). Phenotypic and functional characterization of bone marrow mesenchymal stem cells derived from patients with multiple myeloma. Leukemia.

[111] Corre J., Mahtouk K., Attal M., Gadelorge M., Huynh A., Fleury-Cappellesso S., Danho C., Laharrague P., Klein B., Rème T. (2007). Bone marrow mesenchymal stem cells are abnormal in multiple myeloma. Leukemia.

[112] Bataille R., Chappard D., Marcelli C., Dessauw P., Baldet P., Sany J., Alexandre C. (1991). Recruitment of new osteoblasts and osteoclasts is the earliest critical event in the pathogenesis of human multiple myeloma. J. Clin. Investig..

[113] Alameda D., Saez B., Lara-Astiaso D., Sarvide S., Lasa M., Alignani D., Rodriguez I., Garate S., Vilas A., Paiva B. (2020). Characterization of freshly isolated bone marrow mesenchymal stromal cells from healthy donors and patients with multiple myeloma: Transcriptional modulation of the microenvironment. Haematologica.

[114] Dotterweich J., Schlegelmilch K., Keller A., Geyer B., Schneider D., Zeck S., Tower R.J., Ebert R., Jakob F., Schütze N. (2016). Contact of myeloma cells induces a characteristic transcriptome signature in skeletal precursor cells -Implications for myeloma bone disease. Bone.

[115] Garcia-Gomez A., Rivas J.D.L., Ocio E.M., Díaz-Rodríguez E., Montero J.C., Martín M., Blanco J.F., Sanchez-Guijo F.M., Pandiella A., San Miguel J.F. (2014). Transcriptomic profile induced in bone marrow mesenchymal stromal cells after interaction with multiple myeloma cells: Implications in myeloma progression and myeloma bone disease. Oncotarget.

[116] Schinke C., Qu P., Mehdi S.J., Hoering A., Epstein J., Johnson S.K., van Rhee F., Zangari M., Thanendrarajan S., Barlogie B. (2018). The Pattern of Mesenchymal Stem Cell Expression Is an Independent Marker of Outcome in Multiple Myeloma. Clin. Cancer Res..

[117] Fernando R.C., Mazzotti D.R., Azevedo H., Sandes A.F., Gil Rizzatti E., de Oliveira M.B., Alves V.L.F., Eugênio A.I.P., de Carvalho F., Dalboni M.A. (2019). Transcriptome Analysis of Mesenchymal Stem Cells from Multiple Myeloma Patients Reveals Downregulation of Genes Involved in Cell Cycle Progression, Immune Response, and Bone Metabolism. Sci. Rep..

[118] Lemaitre L., Ferreira L.D.S., Joubert M.-V., Avet-Loiseau H., Martinet L., Corre J., Couderc B. (2020). Imprinting of Mesenchymal Stromal Cell Transcriptome Persists even after Treatment in Patients with Multiple Myeloma. Int. J. Mol. Sci..

[119] de Jong M.M.E., Kellermayer Z., Papazian N., Tahri S., Bruinink D.H.O., Hoogenboezem R., Sanders M.A., van de Woestijne P.C., Bos P.K., Khandanpour C. (2021). The multiple myeloma microenvironment is defined by an inflammatory stromal cell landscape. Nat. Immunol..

[120] Garayoa M., Garcia J.L., Santamaria C., Garcia-Gomez A., Blanco J.F., Pandiella A., Hernández J.M., Sanchez-Guijo F.M., del Cañizo M.C., Gutiérrez N.C. (2009). Mesenchymal stem cells from multiple myeloma patients display distinct genomic profile as compared with those from normal donors. Leukemia.

[121] Garcia-Gomez A., Li T., de la Calle-Fabregat C., Rodríguez-Ubreva J., Ciudad L., Català-Moll F., Godoy-Tena G., Martín-Sánchez M., San-Segundo L., Muntión S. (2021). Targeting aberrant DNA methylation in mesenchymal stromal cells as a treatment for myeloma bone disease. Nat. Commun..

[122] Soekojo C.Y., Chng W.J. (2022). Treatment horizon in multiple myeloma. Eur. J. Haematol..

[123] Kristensen I.B., Pedersen L., Rø T.B., Christensen J.H., Lyng M.B., Rasmussen L.M., Ditzel H.J., Børset M., Abildgaard N. (2013). Decorin is down-regulated in multiple myeloma and MGUS bone marrow plasma and inhibits HGF-induced myeloma plasma cell viability and migration. Eur. J. Haematol..

[124] Yaccoby S., Wezeman M.J., Zangari M., Walker R., Cottler-Fox M., Gaddy D., Ling W., Saha R., Barlogie B., Tricot G. (2006). Inhibitory effects of osteoblasts and increased bone formation on myeloma in novel culture systems and a myelomatous mouse model. Haematologica.

[125] Zavrski I., Krebbel H., Wildemann B., Heider U., Kaiser M., Possinger K., Sezer O. (2005). Proteasome inhibitors abrogate osteoclast differentiation and osteoclast function. Biochem. Biophys. Res. Commun..

[126] Terpos E., Heath D.J., Rahemtulla A., Zervas K., Chantry A., Anagnostopoulos A., Pouli A., Katodritou E., Verrou E., Vervessou E.C. (2006). Bortezomib reduces serum dickkopf-1 and receptor activator of nuclear factor-kappaB ligand concentrations and normalises indices of bone remodelling in patients with relapsed multiple myeloma. Br. J. Haematol..

[127] Terpos E., Kastritis E., Ntanasis-Stathopoulos I., Christoulas D., Papatheodorou A., Eleutherakis-Papaiakovou E., Kanellias N., Fotiou D., Ziogas D.C., Migkou M. (2019). Consolidation therapy with the combination of bortezomib and lenalidomide (VR) without dexamethasone in multiple myeloma patients after transplant: Effects on survival and bone outcomes in the absence of bisphosphonates. Am. J. Hematol..

[128] Hurchla M.A., Garcia-Gomez A., Hornick M.C., Ocio E.M., Li A., Blanco J.F., Collins L., Kirk C.J., Piwnica-Worms D., Vij R. (2013). The epoxyketone-based proteasome inhibitors carfilzomib and orally bioavailable oprozomib have anti-resorptive and bone-anabolic activity in addition to anti-myeloma effects. Leukemia.

[129] Terpos E., Ntanasis-Stathopoulos I., Katodritou E., Kyrtsonis M.-C., Douka V., Spanoudakis E., Papatheodorou A., Eleutherakis-Papaiakovou E., Kanellias N., Gavriatopoulou M. (2021). Carfilzomib Improves Bone Metabolism in Patients with Advanced Relapsed/Refractory Multiple Myeloma: Results of the CarMMa Study. Cancers.

[130] Cata J.P., Weng H.-R., Burton A.W., Villareal H., Giralt S., Dougherty P.M. (2007). Quantitative sensory findings in patients with bortezomib-induced pain. J. Pain.

[131] Xiao Y., Yin J., Wei J., Shang Z. (2014). Incidence and risk of cardiotoxicity associated with bortezomib in the treatment of cancer: A systematic review and meta-analysis. PLoS ONE.

[132] Richardson P.G., Briemberg H., Jagannath S., Wen P.Y., Barlogie B., Berenson J., Singhal S., Siegel D.S., Irwin D., Schuster M. (2006). Frequency, characteristics, and reversibility of peripheral neuropathy during treatment of advanced multiple myeloma with bortezomib. J. Clin. Oncol..

[133] Wang Q., Dong Z., Su J., Huang J., Xiao P., Tian L., Chen Y., Ma L., Chen X. (2021). Ixazomib inhibits myeloma cell proliferation by targeting UBE2K. Biochem. Biophys. Res. Commun..

[134] Tibullo D., Longo A., Vicario N., Romano A., Barbato A., Di Rosa M., Barbagallo I., Anfuso C.D., Lupo G., Gulino R. (2020). Ixazomib Improves Bone Remodeling and Counteracts sonic Hedgehog signaling Inhibition Mediated by Myeloma Cells. Cancers.

[135] Garcia-Gomez A., Quwaider D., Canavese M., Ocio E.M., Tian Z., Blanco J.F., Berger A.J., Ortiz-de-Solorzano C., Hernández-Iglesias T., Martens A.C. (2014). Preclinical activity of the oral proteasome inhibitor MLN9708 in Myeloma bone disease. Clin. Cancer Res..

[136] Diaz-Delcastillo M., Gundesen M.T., Andersen C.W., Nielsen A.L., Møller H.E.H., Vinholt P.J., Asmussen J.T., Kristensen I.B., Nyvold C.G., Abildgaard N. (2023). Increased Bone Volume by Ixazomib in Multiple Myeloma: 3-Month Results from an Open Label Phase 2 Study. J. Bone Miner. Res..

[137] Terpos E., Ntanasis-Stathopoulos I., Kastritis E., Hatjiharissi E., Katodritou E., Eleutherakis-Papaiakovou E., Verrou E., Gavriatopoulou M., Leonidakis A., Manousou K. (2022). Daratumumab Improves Bone Turnover in Relapsed/Refractory Multiple Myeloma; Phase 2 Study “REBUILD”. Cancers.

[138] Rogers M.J., Crockett J.C., Coxon F.P., Mönkkönen J. (2011). Biochemical and molecular mechanisms of action of bisphosphonates. Bone.

[139] van de Donk N.W., Usmani S.Z. (2018). CD38 Antibodies in Multiple Myeloma: Mechanisms of Action and Modes of Resistance. Front. Immunol..

[140] Vasiliadis E.S., Evangelopoulos D.-S., Kaspiris A., Benetos I.S., Vlachos C., Pneumaticos S.G. (2022). The Role of Sclerostin in Bone Diseases. J. Clin. Med..

[141] Gandolfi S., Laubach J.P., Hideshima T., Chauhan D., Anderson K.C., Richardson P.G. (2017). The proteasome and proteasome inhibitors in multiple myeloma. Cancer Metastasis Rev..

[142] Dadzie T.G., Green A.C. (2022). The role of the bone microenvironment in regulating myeloma residual disease and treatment. Front. Oncol..

[143] Khoo W.H., Ledergor G., Weiner A., Roden D.L., Terry R.L., McDonald M.M., Chai R.C., De Veirman K., Owen K.L., Opperman K.S. (2019). A niche-dependent myeloid transcriptome signature defines dormant myeloma cells. Blood.

[144] Lawson M.A., McDonald M.M., Kovacic N., Khoo W.H., Terry R.L., Down J., Kaplan W., Paton-Hough J., Fellows C., Pettitt J.A. (2015). Osteoclasts control reactivation of dormant myeloma cells by remodelling the endosteal niche. Nat. Commun..

